# Vector control for *Aedes aegypti* and *Aedes albopictus* mosquitoes implemented in the field in sub-Saharan Africa: A scoping review

**DOI:** 10.1371/journal.pntd.0013203

**Published:** 2025-07-09

**Authors:** Luciana Lepore, Veerle Vanlerberghe, Kristien Verdonck, Emery Metelo, Mawlouth Diallo, Wim Van Bortel

**Affiliations:** 1 Department of Public Health, Institute of Tropical Medicine, Antwerp, Belgium; 2 Entomology Department, National Institute of Biomedical Research, Kinshasa, Democratic Republic of the Congo; 3 Medical Entomology Pole, Institut Pasteur de Dakar, Dakar, Senegal; 4 Department of Biomedical Sciences, Institute of Tropical Medicine, Antwerp, Belgium; University of California Davis School of Veterinary Medicine, UNITED STATES OF AMERICA

## Abstract

**Background:**

*Aedes*-borne diseases are increasingly reported in sub-Saharan Africa (SSA), where evidence on *Aedes* control remains scarce. This study reviews *Aedes* vector control (VC) interventions evaluated in field settings in SSA, to aid future VC strategies.

**Methodology:**

Literature was searched via PubMed and ISI Web of Knowledge, using a broad search strategy based on *Aedes* species and SSA countries. Two reviewers independently screened all records by title/abstract and full text. The evidence was described, discerning between integrated VC strategies during outbreaks and single interventions in non-outbreak settings. A critical assessment of the quality of evidence was provided.

**Principal findings:**

We included 8 studies evaluating 7 interventions (2 studies referred to the same intervention). The studies were heterogeneous in study design, VC methods, and outcome measurement. Four studies were from West Africa. The studies reported on integrated (2/8) and single (6/8) interventions, including three cluster randomized controlled trials. Non-chemical methods targeting immature stages were frequently reported (5/7), followed by chemical methods against adult (4/7) and immature stages (2/7). Community-based environmental management for source reduction (4/7) showed improved knowledge, but did not lead to a change in practical behavior and/or reduction in entomological indices. Chemical methods were reported to have a temporary impact on entomological indices. Most studies (7/8) used entomological indices, only one in combination with epidemiological outcomes. The assessment of quality of evidence revealed some shortcomings in study designs and issues related to epidemiological outcomes, post-intervention follow-up duration, entomological sampling procedures.

**Conclusions:**

Compared with Latin America and Asia, the studies evaluating *Aedes* VC interventions in SSA are limited. A temporary impact of chemical interventions was reported on entomological parameters, but evidence for reduced transmission was lacking. Environmental management strategies involving communities deserve further study, particularly when integrated with other VC measures tailored to vector bionomics.

## Author summary

To date, solid evidence on the effectiveness of vector control methods against *Aedes* mosquitoes is scarce, with most research coming from Latin America and Asia, although arboviruses are known to circulate in sub-Saharan Africa as well. Our scoping review aims to describe the interventions against *Aedes* mosquitoes implemented to date in sub-Saharan Africa and their field-tested effectiveness, highlighting the strengths and limitations of available research to provide insights for improving future vector control strategies. We found that the literature in this field is limited and heterogeneous, thus leaving a critical knowledge gap on effectiveness of *Aedes* vector control interventions in sub-Saharan African countries. Although data are scarce and study design are variable, chemical interventions, applied alone or as part of integrated vector control strategies, have demonstrated some benefits. However, these effects have been short-term and subject to failure if not tailored to how the *Aedes* mosquito behaves in a specific environment. Community-based interventions aimed at reducing mosquito breeding sources have been investigated in sub-Saharan Africa, but lack sufficient community perception analysis and process evaluation to assess their full impact.

## Introduction

*Aedes aegypti* and *Aedes albopictus* mosquitoes are competent vectors for several arboviruses, including dengue (DEN), yellow fever (YF), chikungunya (CHIK) and Zika (ZIK), which significantly impact human health. The year 2023 witnessed the effects of the El Niño phenomenon leading to a global surge in dengue cases, with simultaneous outbreaks worldwide [1]. In the same year, an increase in autochthonous dengue cases was recorded in the European mainland, with even higher numbers recorded in 2024 [2]. Over the past five years, the increase in dengue cases has been particularly pronounced in the Region of the Americas, with Brazil having the highest burden [3].

In sub-Saharan Africa (SSA), arboviruses have been reported to circulate silently and actively [4], contributing to acute non-malarial febrile illnesses, with dengue and chikungunya being among the most common [5,6]. Although there is increasing evidence of *Aedes*-transmitted disease in SSA, based on sero-surveys [7] and outbreak investigations [8], information on their epidemiology remain fragmented. Surveillance in this region largely focuses on outbreaks, and deaths caused by YF continue to occur despite the availability of an effective vaccine [7]. The World Health Organization (WHO)’s 2023 report on the global dengue situation lists Africa among the top four regions most affected by arboviral diseases. Outbreaks have been reported in 15 of the 47 African countries [1], and dengue is potentially endemic in at least 34 African nations [9]. The same year, Burkina Faso experienced a dengue epidemic with 70.433 probable cases and 709 recorded deaths [10]. Mathematical models further suggest that the burden of dengue in Africa is comparable to that in the Americas, while the disease receives considerably less attention [9]. The discrepancy between reported cases and model prediction may be due in part to the nonspecific symptoms of *Aedes*-transmitted diseases, which often mimic malaria leading to misdiagnosis in the absence of arbovirus-specific diagnostic tests.

Besides vaccines for YF that are existing since a long time, recently new vaccines are becoming available against *Aedes*-transmitted diseases, such as dengue and chikungunya [11]. Further investigation is needed into vaccine efficacy and their contribution as a component of control programmes. Moreover, proper clinical management of human cases remains a challenge. Thus, preventing transmission through effective surveillance and vector control (VC) strategies is crucial to reduce the disease burden and to avert severe public health consequences. The presence of *Aedes* mosquitoes in SSA is well documented, with *Ae. aegypti* and *Ae. albopictus* prevalent in urban and peri-urban environments [12]. However, there is growing evidence of their genetic and phenotypic diversity in SSA, compared to Asia and Latin America, necessitating the acquisition of continent-specific knowledge on their bionomic features [13]. Taken together with context-specific knowledge of resistance patterns to larvicides and adulticides, this is fundamental for the development of VC strategies [14–16].

Evidence on the effectiveness of control methods against *Aedes* mosquitoes in SSA remains scarce. Systematic reviews of *Aedes* mosquito control interventions have largely focused on studies from Latin America and Asia [17–19], with little to no data on VC tools used in the African context. A recent systematic review on household-level interventions against *Aedes* includes examples from three countries in SSA [20] but, overall, this region is underrepresented in the literature compared to research on *Anopheles* mosquitoes, where SSA has historically played a significant role.

As a comprehensive synthesis of what has been conducted so far in SSA is lacking, and taking into account the relevance of adapting VC tools to local conditions, our review aims to assess evidence on the effectiveness of interventions targeting *Ae. aegypti* and *Ae. albopictus* in field settings in SSA. We also seek to understand how these have been deployed and evaluated, with the goal of informing future programmatic efforts for vector-oriented preventive control.

## Methods

### Objectives

This study reviews the literature on *Ae. aegypti* and *Ae. albopictus* VC in the SSA context, focusing on three main objectives: a) describing the tools, interventions, and strategies implemented and evaluated in SSA and published since 2000, b) summarize the effectiveness reported by the authors of VC tools evaluated in the field; and c) comparing the entomological and/or epidemiological outcomes reported in the selected studies. Due to the broad scope and anticipated heterogeneity in study types, interventions, and outcomes, a scoping review approach was chosen. This review follows the guidelines of the Preferred Reporting Items for Systematic reviews and Meta-Analyses extension for Scoping Reviews (PRISMA-ScR) (S1 PRISMA Checklist). The original search was conducted in March 2023, with an updated search on March 1^st^ 2024. The review protocol is registered on the Zenodo repository (https://doi.org/10.5281/zenodo.8010539).

### Search strategy and inclusion criteria

We used the SPICE framework to frame the study question, defining the study’s setting, population/perspective, intervention, comparison and evaluation criteria (Table 1) [21].

**Table 1:** Overview of the key elements of the scoping review question following the SPICE framework.

Key Elements	Definition
Setting	Interventions implemented in the field since 2000 in Sub-Saharan Africa. Sub-Saharan Africa is defined as per the WHO Regional Office for Africa -Country list.
Population/Perspective	*Aedes aegypti* and *Aedes albopictus* mosquitoes.
Intervention	Any tool, strategy, or intervention aimed at reducing the presence or abundance of *Aedes* mosquitoes or the incidence of *Aedes*-transmitted diseases.
Comparison	Subgroups with and without intervention, and/or before and after intervention.
Evaluation	Any data reporting entomological indices (including but not limited to density, infection rate, longevity, man-vector contact) and/or any epidemiological data on human infections (including, but not limited to disease incidence) related to *Aedes*-borne diseases.

The search strategy included keywords identified based on (i) the specific mosquito populations targeted by the interventions, *Ae. aegypti* and *Ae. albopictus*, including alternative names, and (ii) the geographical context of the interventions, i.e., the SSA countries listed by the WHO Regional Office for Africa (S1 Table). Boolean operators were used to combine the keywords appropriately. A decision was made for a broad and sensitive search strategy without restricting the search by study design in an attempt to include the different VC study designs for which we anticipated a lack of standardized and consistent terms for a literature search. The search was conducted across PubMed and ISI Web of Knowledge. Additionally, a snowballing technique was applied, reviewing reference lists of relevant articles and systematic reviews to identify any further studies that met the inclusion criteria, but were not captured in the initial systematic search. Google Scholar was consulted for the first 100 records and compared to the systematic search as validity check.

As this review focuses on studies evaluating the effectiveness of VC tools in the field, we selected records that (i) were published from 01/01/2000 onward, (ii) were primary study reports or review articles, (iii) focused on evaluating one or more *Ae. aegypti* and/or *Ae. albopictus* VC intervention(s), (iv) described an intervention implemented in the field in SSA, and (v) provided a measure of entomological and/or epidemiological outcomes. Studies exclusively reporting on laboratory experiments, proofs of concept, or those focused solely on community acceptability were excluded. For the purposes of this scoping review, opinion articles and commentaries were also excluded. No *a priori* restrictions were applied concerning language or study design. A checklist with exclusion criteria was used to assist reviewers in screening and selecting relevant papers (S2 Table). This checklist resulted from calibration based on the screening of the first 20 records retrieved from the initial literature search.

### Study records: collection, selection and data extraction

All records were exported to the web-based Covidence software, which was used for de-duplication, title/abstract screening, and selection based on full-text evaluation. Two reviewers (LL and WVB) independently screened all papers based on title and abstract first and full text subsequently. Doubtful records at title/abstract screening, were included for full-text screening in which disagreements were discussed between the two reviewers and, if no agreement was reached, a senior academic researcher was available for consultation. Entomological research experts from two SSA countries (EM and MD) were consulted to revise and agree on the list of included records, and to ensure no relevant articles were missed. Data extraction was performed by one reviewer (LL), using a table to record the following variables: study record, location, objectives, study design, intervention, outcome, impact evaluation, limitations, strengths and conclusions. A second reviewer (WVB) cross-checked each entry. Any doubts were discussed until consensus was reached, or a senior researcher was consulted to resolve differences.

### Data synthesis

The process of literature search and selection was reported through a PRISMA flowchart, indicating the reasons for exclusion. The main characteristics of the studies were provided and a comprehensive description of the evidence was performed. Studies conducted during outbreaks and evaluating the effectiveness of an integrated VC strategy, using multiple VC methods, were described separately from those conducted outside epidemic settings assessing single VC interventions. The latter were classified and described according to the method used (chemical or non-chemical, against mature or immature *Aedes* stages). Finally, a critical appraisal of the quality of the evidence was provided looking at the design features of relevance for a good study design for evaluating the effectiveness of VC interventions based on what has been previously discussed by Wilson et al. in this regard [22].

## Results

### Selection and key characteristics of sources of evidence

**Fig 1:**
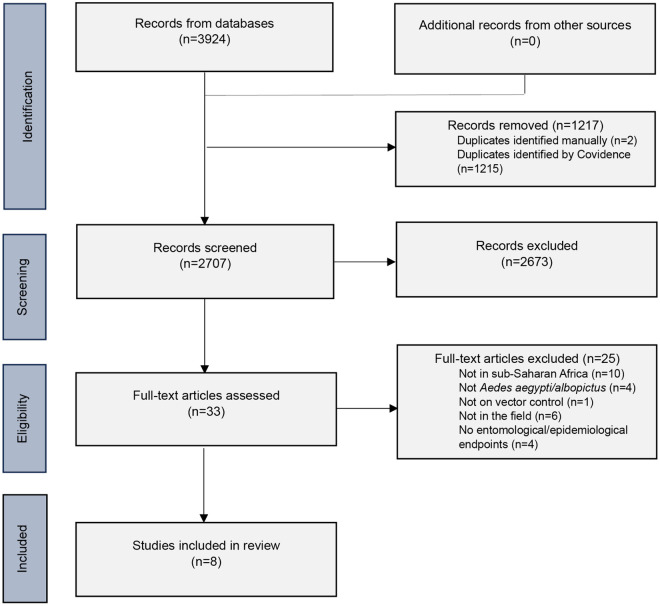
PRISMA flowchart of the scoping review.

The original search identified 3924 records. After removing duplicates, 2707 records were screened by title and abstract, of which 33 remained for full-text screening and eight studies were finally included for data extraction and analysis (Fig 1).

Four studies originated from West Africa, with three from Burkina Faso and one from Côte d’Ivoire. The remaining four studies were conducted in Ethiopia, Gabon, Kenya, and Sudan. As two of the records from Burkina Faso reported on two consecutive analyses of the same intervention, this review comprises 8 studies evaluating 7 interventions. The earliest paper was published in 2005, while the other seven were published between 2016 and 2022.

VC intervention evaluations were as follows: three cluster-randomized controlled trials (RCT), three before-and-after studies, one retrospective cohort analysis, and one case-study. Two of the three before-and-after studies were carried out during outbreaks, specifically during a dengue outbreak in Sudan in 2010 and a chikungunya outbreak in Ethiopia in 2019. The remaining (6/8) referred to studies realized outside of *Aedes-*borne disease outbreaks.

Most of the interventions (5/7) focused on non-chemical methods targeting the larval or pupal stages of mosquitoes, followed by chemical methods targeting adult stages (4/7) and chemical methods targeting immature stages (2/7). There were no studies evaluating non-chemical methods targeting adult *Aedes* mosquitoes, such as mass trapping or *Wolbachia*. Community participation was included in four of the seven interventions. Integrated VC strategies, combining both chemical and non-chemical VC measures, were employed in the two studies performed during outbreaks. Entomological endpoints were assessed in seven studies, and epidemiological outcomes in only two. Two studies including community participation, assessed also knowledge and behavior, with one study integrating both qualitative and quantitative methods (Table 2). The key characteristics of the included studies are presented in Table 2 and more extensively in the Supplementary material (S3 Table), detailing study objectives, methodology, interventions, indicators and main findings.

**Table 2:** Key characteristics of the included studies.

Paper	First author Year		*Seidahmed* *2012* [23]	Waldetensai 2021 [24]	Ouédraogo 2018 [25]	*Bonnet* *2020* [26]	Forsyth 2022 [27]	*Dambach* *2021* [28]	*Gabor* *2016* [29]	*Kone* *2005* [30]
**Country**		*Sudan*	*Ethiopia*	*Burkina Faso*	*Burkina Faso*	*Kenya*	*Burkina Faso*	*Gabon*	*Côte d’Ivoire*
Study Design	Cluster-RCT									
	Before-and-after study									
	Retrospective cohort									
	Case study^*^									
Outbreak setting										
Intervention	Chemical	Immature	*temephos*	*temephos*						
	Chemical	Adult	*permethrin* *LLINs* *Topical repellents*	*propoxur*					*ITNs*	*deltamethrine*
	Non chemical	Immature	*Environ managem*	*Environ managem*	*Environ managem*		*Environ managem*	*Bti*		
Community participation										
Outcome	Entomological indices									
	Epidemiological indices									
	Knowledge									
	Behavior									
Qualitative assessment										

Legend: Bti, Bacillus thuringiensis israelensis – Environ managem, Environmental management– ITN, Insecticide-treated net – LLIN, Long lasting insecticidal nets – RCT, Randomized Controlled Trial

* based on a cluster-RCT

Grey boxes indicate the presence of the variable, while white boxes indicate its absence.

## Results of individual sources of evidence

### Interventions in outbreak settings

Both articles addressing outbreak settings presented an integrated response using multiple VC methods, and are, therefore, discussed separately, as they evaluate the effectiveness of a combined strategy rather than individual interventions.

#### Dengue outbreak in Sudan in 2010.

During the 2010 dengue outbreak in Port Sudan city, the implementation of an integrated strategy was assessed through a before-and-after study [23]. This strategy combined community mobilization, focal space spraying consisting of indoor and outdoor thermal fogging and ultra-low volume (ULV) sprays of permethrin, and chemical larviciding of outdoor potential breeding sites with temephos. Active source reduction was carried out by trained health workers, along with communal space and household inspections by community volunteers, covering over 70% of the houses of Port Sudan city. Health education campaigns promoted water filtering and scrubbing and covering of clay pots and barrels. Long-lasting insecticidal nets (LLINs) were distributed to inpatients and outpatients, and the use of repellents during the daytime was encouraged. The strategy was implemented over a 12-week period in areas with a high number of dengue cases (transmission foci). The authors documented a significant decrease in entomological indices over 14 weeks of vector surveys, and a reduction in dengue transmission to a zero incidence about 19 weeks after the launch of the dengue vector action plan.

Vector surveillance was realized in eight sentinel sites across the eastern, middle and southern sectors of the city. A total of 22% of the indoor water storage containers inspected, were infested with *Ae. aegypti* larvae and/or pupae: mainly in clay pots (75%) and plastic barrels (15%). Entomological and epidemiological indices were compared before and after the intervention during the outbreak period showing pronounced reductions: House index (HI) decreased from 100% to 16% (F = 57.8, P < 0.001) and pupal/person index (P/PI), from 0.77 to 0.10 (F = 3.06, P < 0.01) while dengue incidence rate dropped from 9 cases/10.000 inhabitants to zero. A regression analysis revealed a significant association between entomological parameters and dengue incidence (R2 = 0.83, F = 23.9, P < 0.001).

#### Chikungunya outbreak in Ethiopia in 2019.

The response to the chikungunya outbreak in Ethiopia in 2019 was assessed through a before-and-after study in eight urban kebeles (corresponding roughly to neighborhood) in the city of Dire Dawa, where one hundred households were selected per kebele, with simple random sampling techniques. The intervention consisted of a multifaceted vector control strategy that included chemical indoor and outdoor space spraying with propoxur, larviciding using temephos in water storage containers, environmental management, and community awareness campaigns and mobilization for active participation in larval source management. This comprehensive approach was implemented over eight weeks [24].

The preferred resting locations for adult *Aedes* mosquitoes were identified as open empty barrels and bedrooms (P < 0.04). Outdoor containers filled with clean rainwater showed the highest infestation rates, with tyres being the most infested (25.2%), followed by barrels (17.8%), flower pots (16.9%) and jerrycans (15.1%). In an evaluation performed before and after one week of implementing mosquito control interventions, the authors documented a reduction in all entomological indices, which, however, still indicated a high probability for arbovirus transmission after the intervention. Container index (CI) decreased from 92.9% to 14.7%, Breteau index (BI) from 141 to 20.1, HI from 90.1% to 7.4% and PI from 1431.4 to 4.12. The authors reported a significant decrease in the number of positive containers with *Aedes* mosquito’s immature stages after the intervention, in comparison to pre-intervention (Kruskal–Wallis test, P < 0.01). Although *Aedes* were still observed in the empty open barrels after chemical spraying, their abundance drastically decreased after control interventions (P = 0.031).

### Interventions outside outbreak settings

The remaining included articles evaluated a single VC method.

#### Environmental management – Non-chemical for *Aedes* immature stages.

Three studies described environmental management implementation alone: two from Burkina Faso, both referring to the same intervention in 2016 [25,26], and one from Kenya in 2017 [27]. In both countries, despite the improvements in community knowledge and self-reported behaviors following these interventions, the studies failed to demonstrate a strong entomological impact.

The intervention implemented in Burkina Faso was a community-based intervention assessed through a cluster-RCT in the city of Ouagadougou, where two comparable neighborhoods were selected and randomly allocated to intervention and control, respectively in Tampouy and Juvenat communities. A total number of 287 and 289 households were randomly sampled in the intervention and control arms, respectively. Entomological indices (primary outcomes) were evaluated at compound level (≥1 households sharing the same living space) while secondary outcomes related to knowledge, attitudes and practices were evaluated at the household level. The community-based intervention included environmental management and community education for behavior change implemented for a period of around 16 weeks concomitant with the dengue transmission peak. Researchers and entomologists experienced in community-based intervention, promoted a participatory process involving and training community members and leaders. Amongst communication activities, a theater group informed the community on dengue transmission, prevention, clinical characteristics, and mosquito breeding sites control. Door-to-door visits, school education, and self-awareness assessment sessions aimed at strengthening community awareness. Entomological outcomes, immunological biomarkers for exposure to mosquito bite and human behaviors and knowledge were assessed [25], together with an in-depth spatial analysis [26]. Regression analysis reported by the authors showed that the intervention resulted in a reduction of exposure to *Ae. aegypti* mosquito bites (coefficient –0.08 [95% CI –0.11 to –0.04]) in the intervention arm post-intervention in comparison to pre-intervention and controlling for trend in the control group. A significant beneficial impact was reported on behavior and knowledge when comparing intervention with control arm: self-reported actions against mosquitoes RR 1.42 [95% CI 1.29–1.57], bed nets use RR 1.31 [95% CI 1.22–1.42], increase in dengue knowledge RR 1.13 [95% CI 1.01–1.27] and identification of appropriate dengue symptoms RR 1.44 [95% CI 1.22–1.69]. Discarded containers were most infested by *Aedes* in both intervention and control areas (62.4% and 68.8%, respectively), followed by water containers (36.5% and 27.5%, respectively). In the comparison intervention-control arms, the intervention reduced the number of *Aedes* immature stages (t = 2.36; P = 0.019) and the within-household average change of the proportion of pupae positive containers was 9.67% (95% CI: 1.1–18.3%) indicating a potential impact of the intervention as reported by the authors. In the intervention area, there was an impact on larval population in the water storage containers (- 69.4%) but not on the discarded ones (+ 5.7%). Ouédraogo et al. showed by a regression model that although CI, HI, BI, P/PI were all reduced in the intervention arm compared to the control arm, the community-based intervention did not show a significant effect on the absolute number of *Ae. aegypti* breeding sites or on the number of larvae and pupae at the compound level [25]. In the subsequent spatial analysis, the authors showed the effect on the concentration of breeding sites in the households: in the intervention arm, the intervention successfully suppressed high and low concentration areas of pupae, whose distribution became more random post-intervention, while clusters persisted in the control arm [26].

Environmental management implemented in Kenya through a community-based intervention focused on source reduction [27] and evaluated through a pair-matched cluster-RCT in 10 coastal villages (=clusters). Matching was based on proximity and similarities in rural/peri-urban status, and village pairs were randomized to either the control (5 villages) or intervention (5 villages) group. Within each village, 60 children, and related caregiver, were randomly selected from the school roster. Primary outcomes related to knowledge and behaviors were assessed from 259 and 261 subjects in the intervention and control arms respectively at baseline, 237 and 247 subjects at 3 months, and 232 and 241 subjects at 12 months evaluation. Entomological indices (secondary outcomes) were assessed in 232 and 248 households in the intervention and control respectively at baseline, and in 233 and 248 at 12 months evaluation. The intervention included covering containers, removing trash and unused containers, moving containers out of the rain, and removing or poking holes in tires. A 3-day workshop with stakeholders was organized by the research team to collaboratively design the intervention aiming at empowering children aged 10–16 years and caregivers, mainly female heads of household, in source reduction practices. The intervention was piloted and refined and thereafter implemented for a period of 12 weeks. Children and their parents were invited to participate in a container cleaning and plastic recycling event and to collect and reuse containers with no immediate purpose. After the final evaluation, the intervention was also implemented in the control villages.

In the assessment of self-reported behavior, the intervention group showed an improvement over the control group (adjusted risk difference of 0.58, 95% CI [0.43 to 0.73]) with the two most commonly reported behaviors being *“covering containers”* and *“moving containers from the rain”*. This was not confirmed in the assessment of observed behaviors where the variable *“at least 1 covered container”* showed no significant difference between the two arms at 12 months of evaluation (adjusted risk difference of -0.08, 95% CI [-0.13, -0.01]). In the intervention group, authors described an improvement in knowledge, reaching >50% at 12 months evaluation (adjusted risk difference 0.69, 95% CI [0.56 to 0.82]). “*Intention to cover*” was reported to be the most frequent behavior and “*moving containers*” was more common among adopters of the intervention. Some barriers to successful implementation were outlined, including interference by others, large number of containers, and loss of covers, while the need to keep water clean and safe and interest in disease prevention were some of the facilitators in the implementation of the community-based intervention. No significant differences were observed between the two arms when comparing pre- and post-intervention evaluations of epidemiological indices (CI adjusted mean difference -0.01, 95% CI [-0.04, 0.02] and HI adjusted risk difference 0.01, 95% CI [-0.03, 0.06]). At the 12-month evaluation, the majority of immature mosquitoes in the intervention arm were found in containers used for laundry (47%), followed by containers with no purpose (39%), and those for sanitation (11.7%). In the control arm, containers with no purpose were reported to be the most infested (71.6%), followed by those for laundry (28.3%).

#### *Bacillus thuringiensis israelensis* (*Bti*) – Non-chemical for *Aedes* immature stage.

The impact of larviciding with *Bti* on *Aedes* mosquitoes was evaluated in a cluster-RCT during larviciding interventions against malaria vectors over a two year-period, starting in 2014, in Burkina Faso [28]. A total number of 127 rural villages were grouped in nine clusters and equally divided into three different ecozones based on similar ecological characteristics. Larviciding options were randomly assigned to the clusters, ensuring that each ecozone included the three study arms. An untreated control arm was compared to *Bti* application of all breeding sites in public spaces (arm *Bti*-100%) and risk map-based *Bti* application (arm *Bti*-50%), one year after the start of the intervention. Adult mosquito monitoring was performed in three central households per study village, for a total number of 27 and 36 villages, before and after the intervention respectively; the semi-urban town of Nouna was added later. *Aedes* capture was reported to be predominantly indoors being 57% (p = 0.071) of the collection and the proportion of *Aedes* collected among all mosquitoes remained unchanged, being 19% (n = 2317) pre-intervention (2013) in four months of sampling vs 22% (n = 5357) in six months of sampling in 2014. The regression analysis showed a significant reduction in *Aedes* mosquito abundance by 34% (vs 70% reduction of *Anopheles*) in the full treatment arm (rate ratio RR 0.66, 95% CI: 0.57–0.76) but not in the risk map-based treatment (RR 0.94, 95% CI: 0.85–1.05). The major impact was identified in the semi-urban town of Nouna and was heterogeneous over time, with a higher impact during August.

#### Insecticide-treated nets (ITNs) – Chemical for *Aedes* adult stage.

The potential influence of insecticide-treated bed nets (ITNs) on dengue and chikungunya seroprevalence was estimated retrospectively in a cohort of neonates previously enrolled in a RCT on the prophylactic use of sulfadoxine/pyrimethamine for malaria in Gabon from December 2002 to April 2007 [29]. Coverage of insecticide treatment of mosquito bed nets was not specified in the paper. Over a 30-month observation period, ITNs usage decreased from 96% to 79%, while seroprevalence for dengue increased from 1.2% to 12.3%. Only one sample was positive for chikungunya (0.6%). Although this was not an objective of the principal study, no significant correlation between ITNs use and dengue and/or chikungunya seropositivity was reported.

#### Spatial spraying – Chemical for *Aedes* adult stage.

A study conducted in Côte d’Ivoire evaluated in 1997 the effect of spatial spraying with two ULV applications of deltamethrin, spaced one week apart, on *Ae. aegypti* [30]. The intervention took place in two different municipalities, a maritime (Port-Bouët) and a forest area (Yopougon), and evaluated in a before-and-after study without a control group. Indoor and outdoor human landing captures was performed in three households, each in one of three randomly chosen neighbourhoods of each of the two locations. At baseline, a different abundance of *Ae. aegypti* adult mosquitoes was identified in the two areas with 5.58 bites/man/sampling period in the maritime area versus 2.5 bites/man/sampling period in the forest area. After the first ULV, an initial reduction in adult *Ae. aegypti* population was observed in both places, a reduction of 37.5% of *Ae. aegypti* population in the maritime area (*vs* 69% reduction in overall mosquito density) and a reduction of 66% in the forest area (*vs* 15% reduction in overall mosquito density). This was not confirmed in the evaluation carried out five days after the second ULV application revealing adult *Ae. aegypti* abundance returned to pre-intervention values in both municipalities. *Aedes* behavior was found to be different in the two municipalities, being more endophagic in maritime (69%) than in forest (11%) areas, which, according to the authors, could have explained the lesser effect of spatial spraying in the maritime area.

### Assessment of the quality of evidence for VC effectiveness evaluation

Based on the paper by Wilson et al [22] that elaborates on the essence of a good study design for evaluating the effectiveness of VC interventions, a critical assessment on the quality of evidence of the included papers was performed (Table 3). Specifically, this focused on study design, implementation and adherence to the intervention, epidemiological and entomological outcomes. None of the studies scored blue/good on all aspects evaluated.

**Table 3:** Quality of evidence assessment.

		Seidahmed, 2012 Sudan	Waldetensai, 2021 Ethiopia	Ouédraogo, 2018 Burkina Faso	Bonnet, 2020 Burkina Faso	Forsyth, 2022 Kenya	Dambach, 2021 Burkina Faso	Gabor, 2016 Gabon	Kone, 2005 Côte d’Ivoire
Intervention implementation design	Cluster level intervention								
	Contemporaneous control group								
	Random allocation of intervention and control								
	Blinding								
	Random site selection for entomological surveys								
	Sample size calculation specified								
	Follow up period of minimum 1 transmission seasons								
	>1 post-intervention entomological sampling								
Intervention adherence	Assessment of quality of intervention								
	Assessment of coverage of intervention								
	Assessment of users compliance								
Epidemiological outcomes	Disease incidence/mortality								
	Seroprevalence								
Entomological outcomes besides traditional larval indices	Automated sampling tools (vector traps)								
	Disease transmission-related entomological outcomes								
	Pupal/person index								
	Adult vector density								

Dark Blue-Present and/or reported

Light Blue-Partially present

Orange-Not present and/or not reported

Only three studies were cluster-RCTs, among them one only included one intervention and one control cluster. All but two studies employed an intervention allocated to clusters and in four cases the intervention was randomly allocated to an intervention group and compared to a contemporaneous control group. In six studies, randomization was also applied for the site selection for entomological indicators monitoring. Sample size calculation was specified in six studies. In five studies the duration of follow up was considered appropriate being at least one transmission season or a one-year period if the transmission is perennial. In only three studies, the entomological sampling was performed more than once during the post-intervention phase. Budget constraints were mentioned in one paper as a limiting factor to have an adequate follow-up period. Blinding was not possible as expected, with the exception of the only study with a qualitative component in which interviewers were blinded to adopter status.

All but three of the papers put in place quality controls of implementation through supervision, random checking, and evaluation of proper application of insecticides. The same studies also assessed intervention adherence through self-reported assessment combined or not with observations, the use of qualitative methods, and use of communication techniques for community motivation and engagement in VC activities.

Regarding the outcomes, only two studies considered epidemiological indicators related to disease incidence and/or infection seroprevalence, providing direct measures of VC interventions impact on human health. In contrast, entomological indicators were consistently used across all studies, offering insights into mosquito population dynamics. Trap-based sampling, a method frequently employed to monitor adult vector density, was used in only one of the three studies assessing adult *Aedes* mosquitoes. This study employed both indoor and outdoor light traps to collect adult mosquitoes. Beyond traditional larval indices, two studies looked at the pupal/person index for immature *Aedes* abundance. Only one study made use of an entomological indicator based on human biomarkers for exposure to *Ae. aegypti* mosquito bite.

## Discussion

Given the increasing global importance of *Aedes*-transmitted diseases, this study aimed to review the literature on the effectiveness of implemented VC methods targeting *Aedes* mosquitoes in SSA. We found only eight studies published since 2000, using various study designs, vector control strategies, and impact estimates. The heterogeneity of included studies limits the strength of conclusions that can be drawn for general recommendation of interventions.

Among the eight studies, three came from Burkina Faso, identified in the WHO Dengue Global Reports as the SSA country most affected by dengue [1,3]. The earliest study, published in 2005, evaluated a chemical intervention carried out in 1997 in Abidjan, Côte d’Ivoire [30]. Arboviruses represent a public health threat in several SSA countries, as evidenced by a major dengue outbreak in 2017 in Côte d’Ivoire [31], and in 2010 in Port Sudan with 3765 cases [23], and by a chikungunya outbreak in 2019 in Dire Dawa, Ethiopia [24], causing 41162 suspected and 16 confirmed cases [32]. Co-circulation of arboviruses has been described, causing simultaneous outbreaks as for example in Gabon [33], and subsequent outbreaks of dengue, chikungunya, Rift Valley fever, YF, and Crimean–Congo hemorrhagic fever in Kenya [34]. If model predictions are correct and the dengue burden in Africa is approximately equivalent to that in the Americas [9], this implies an extremely important gap in knowledge on arbovirus circulation and the huge need for VC effectiveness estimates in African countries. Most of the studies (5/8) were published from 2018 onwards, limiting our ability to assess trends over time.

The VC methods evaluated were varied, including both chemical and non-chemical approaches targeting immature stages, as well as chemical methods for adult *Aedes*. The majority of included interventions (4/7) investigated environmental management strategies including community-based interventions. Environmental management activities varied both in terms of the activities performed and in terms of places and people involved: households versus schools or adults versus children. The community was empowered through education but played a varying role. Often the community was a recipient of the VC intervention, and rarely involved in the intervention’s decision-making pathway. In a previous review by Bowman et al. on dengue VC effectiveness, interventions based on community participation performed significantly better in reducing vector larval development sites [17]. The strongest evidence comes from Cuba where intervention promoted by community groups for environmental management contributed not only to reducing vector indices, but also impacted dengue transmission [35]. More recent studies from Latin America and the Caribbean region also showed that environmental management, especially within integrated strategies, can lead to long-term beneficial effects when community participation goes beyond mere health education campaigns [36]. Conversely, from the examples included in our review, although community-based interventions showed a beneficial increase in knowledge and self-reported beneficial behaviors, they did not lead to a corresponding shift in practical behavior and/or impact on entomological indices. Indeed, in Kenya, the improvement in self-reported behavior against the *Aedes* mosquito did not match with the behaviors observed by the study teams and the community-based intervention did not show an impact on entomological indices [27]. Likewise in Burkina Faso, the community-based intervention had no effect on the absolute number of *Ae. aegypti* breeding sites [25]. Additionally, the integrated VC strategy implemented in Sudan in 2010 highlighted the challenges of maintaining household adherence to regular cleaning activities. It underscored the need for continuous health education programs to effectively promote and sustain community participation [23]. In this regard, it is important to keep in mind the complex nature of community-based interventions [37]. The qualitative approach that was presented in only one of the studies may be beneficial to explain how such interventions succeed or fail and what implementation processes are conducive.

It is worth noting that, in our review, community involvement focused only on the control of immature stages of *Aedes*, missing the opportunity to target interventions against adult mosquitoes that might enhance VC effectiveness by directly impacting the reduction of virus transmission through the reduction of adult mosquito density and longevity [38]. This has also been noted by Montenegro-Quiñonez et al. in their review on *Aedes* VC at the household level, in which they similarly identified only a minor role of the community in interventions against adult mosquitoes [20]. The involvement of the communities could enhance the acceptability and uptake of a VC activity against adult *Aedes* mosquitoes with promising effects [36], for example mass trapping, and increase the sustainability of an integrated *Aedes* VC strategy [39].

As emerged from our review, the same intervention can result in different impacts depending on the context of its implementation, be it in urban, rural, maritime or forest ecosystems. Behavioral differences in the *Aedes* mosquito population have indeed emerged. While in the Ethiopian city of Dire Dawa *Aedes* adult mosquitoes were prevalent in outdoor open empty barrels, in rural Burkina Faso they were mainly caught indoors. In Côte d’Ivoire, overall exophagic behavior prevailed (56%), but in the specific maritime area, mosquitoes showed more clearly endophagic behavior. Although *Aedes* species are generally considered highly anthropophilic, feeding and resting primarily inside human dwellings [40], a more diverse range of behaviors seem indeed to be present in SSA. Activity outside dwellings by *Ae. aegypti* has been reported from West Africa, as in Ghana [41], Senegal [40], and from East and Central Africa, as in the case of the Democratic Republic of the Congo (DRC) [42,43] and Kenya [44]. As previously highlighted by Badolo et al, it is possible that exophagic or endophagic preferences may not be exclusive and coexist in mosquito populations [13]. Preferences for oviposition sites by *Ae. aegypti* also varied in our included papers with indoor containers most infested in Port Sudan, Sudan, while outdoor containers containing rainwater prevailed in Dire Dawa, Ethiopia, and discarded containers in rural Burkina Faso. Prior knowledge of specific mosquito populations bionomics is indeed essential to optimize VC interventions, as is understanding the specific composition of the local vector population. More specifically, in the context of SSA, the subspecies *Ae. aegypti aegypti*, known as the domestic ecotype found outside Africa, coexists with *Ae. aegypti formosus*, which more traditionally occupies forest habitats with zoophagic tendencies and preferences for oviposition in natural rather than artificial sites [45], but which is increasingly being found in urban environments [46]. These divergent behaviours suggest that VC interventions effective in other regions, such as Asia and Latin America, where *Aedes* mosquitoes are typically endophagic and endophilic, might not be as effective if applied in the same way in SSA. This calls for a reflection on the possible limitations of strategies based on residual indoor spraying.

It is worth noting that none of the publications included in our review identified the presence of *Ae. albopictus*, despite it being known as an important vector of *Aedes*-borne diseases in Africa [12,47].

In our review, chemical methods against the adult population of *Aedes* in SSA (permethrin, deltamethrin and propoxur) alone or in combination with larvicides (temephos) were reported to decrease the entomological indicators. In two studies, one based on a chemical adulticide [30] and the other on a biological larvicide [28], researchers reported the short time effect of the intervention on the *Aedes* population, with mosquito densities returning to or even going beyond the starting point, i.e., the rebound effect, as described by Horstick et al [48]. None of the included studies reported on insecticide resistance, however, this is of primary importance when planning and evaluating interventions, given the potential negative effect of insecticide resistance on VC effectiveness. After a long period of inadequate monitoring of *Aedes* resistance to insecticides in SSA, recent studies, particularly from West Africa, are offering new insights on this topic [49]. Overall resistance to DDT has been described as widespread in SSA for both *Ae. aegypti* and *albopictus*, while resistance to pyrethroids (mainly permethrin and deltamethrin) appears to be more sporadic [46,50], which are often used for insecticide treated nets and indoor residual spraying. Resistance to pyrethroids have been reported in Burkina Faso [51], Benin [16], Ghana [41], Senegal [15], DRC [52], Capo Verde [53], Cameroon [54] and contrasting resistance patterns from the Republic of the Congo [55]. In Cote d’Ivoire, where the study based on the use of deltamethrin was carried out, field populations of *Ae. aegypti* were found to be susceptible to deltamethrin in Yopougon but not in Port-Bouët [56]. Conversely, in the Port Sudan area, the other study site included in our review, sensitivity to pyrethroids remained preserved though certainly requiring attention to ensure its future effectiveness [57]. Some resistance to carbamates (usually propoxur) has been described in SSA [46], while biological and chemical larvicides, *Bti* and temephos, seem to maintain a less problematic level of resistance [49]. Considering insecticide resistance that is occurring in several areas of endemic countries in SSA where many insecticides from the organochlorine, organophosphate and pyrethroid groups are also used for the control strategy of anophelines, besides *Aedes* [57], testing new sustainable and innovative technologies for vector control is becoming crucial. These include, amongst others, the *Wolbachia*-based methods, declared by the WHO in 2021 to be an “intervention of public health interest” [58], and based on *Wolbachia*’s ability to manipulate reproduction and reduce the transmission of vector-borne pathogens [59]. Large-scale use of *Wolbachia*-infected *Ae. aegypti* has been deployed in Australia, Asia and the Americas, coinciding with a reduction in the local transmission of dengue. There are currently no pilot studies in SSA [58].

Finally, the integrated VC interventions presented in the included studies were evaluated only during outbreaks, so evidence on the effectiveness of integrated VC strategies against *Aedes* in non-outbreak settings in SSA is lacking. Ideally vector control programs should be integrated, have a pre-rainy season prevention activities, and a strategy for rapid response to disease outbreaks [13,60].

The critical appraisal of the quality of the VC effectiveness in the included studies was based on evidence-based VC considerations reported by Wilson et al. [22]. This assessment aimed to highlight the main strengths and shortcomings of the included articles and provide insights for improving the design and implementation of VC studies. Only three included studies adopted the cluster-RCT approach [25,27,28]. This methodology lends itself to the highest methodological quality in VC evaluation, as opposed to before-and-after studies, but as shown in Table 3, these RCTs had several shortcomings, which limits the generalizability for findings. In four papers [23,24,29,30], the lack of a control group hindered the assessment of impact. This limitation made it difficult to account for seasonal changes, such as variations in precipitation, which could either mask or exaggerate the observed effect [22]. Apart from blinding, which is notoriously difficult in VC studies, the other critical points in most studies were the post-intervention evaluation period, entomological sampling, and the type of outcome used for evaluation. We considered as appropriate a follow-up period of at least one transmission season or a 1-year period if transmission is perennial, indicated as the minimum follow-up duration by Wilson et al. Whether using chemical methods or targeting behavioral change in community-based-type interventions, follow-up periods with repeated measurements are needed to detect sustained behavioral change or maintained intervention effects. In relation to outcomes, almost all studies included entomological indicators. It is worth mentioning that these studies did not rely exclusively on the traditional *Stegomyia* indices only (HI, CI, BI), but in most cases included indices of adult *Aedes* abundance, the pupal indices, human biomarkers for exposure to *Ae. aegypti* mosquitoes. *Stegomyia* indices, which assess the immature mosquito stage (larvae and pupae), were developed over 90 years ago for YF, and their reliability and sensitivity as indicators of dengue epidemic risk have been widely questioned [17,61]. As reported by Wilson et al, measuring pupae and/or adult vector density is more appropriate for assessing transmission risk [22]. From a public health perspective, however, epidemiological outcomes are needed to demonstrate the effectiveness of interventions in protecting human populations [22], which were lacking in most of the included studies. Only the study performed during the dengue epidemic in Sudan examined both entomological and epidemiological indices, where authors identified a significant relationship between the reduction in dengue incidence and entomological outcomes. It should be noted, however, that in both outbreak studies [23,24], the traditional entomological indices, although reduced post-intervention, still indicated a high probability of arbovirus transmission. Providing evidence on effectiveness should include not only entomological indices [61], but also epidemiological information on disease occurrence, based on incidence data, or even better sero-prevalence seen the importance of asymptomatic infections.

## Strengths and limitations

One of the strengths of the present study was the use of a broad search string, which was particularly advantageous given the current unavailability of MESH terms specifically related to the VC field. This approach enabled broad coverage in our scoping review. At the same time, the paucity of the included studies related to SSA published since 2000, not meeting the quality standards, as well as the heterogeneity of the results are among the limitations of our study. None of the systematic and scoping reviews cited in our discussion, except the one by Montenegro-Quiñonez et al [20], considered VC practices against *Aedes* implemented in SSA, a region that remains underrepresented in the literature.

## Conclusion

In conclusion, there are only a limited number of studies evaluating the effectiveness of VC strategies against *Aedes* in SSA compared to what is published in Latin America and Asia. The paucity and heterogeneity in the study designs and evaluation procedures of the included studies, hinder the ability to draw unequivocal conclusions on *Aedes* control methods in SSA, revealing a critical research gap. Chemical interventions against adult or immature stages of *Aedes* (permethrin, deltamethrin, temephos), either alone or as part of an integrated VC strategy, seemed to have a positive, albeit temporary, impact on entomological indicators, however, no evidence was reported on epidemiological outcomes. Additionally, environmental community-based management interventions have been prominent in the studies and warrant further investigation on how to tailor *Aedes* VC strategies to local vector bioecology, but also to community and individual behaviors.

## Supporting information

S1 PRISMA Checklist Preferred Reporting Items for Systematic reviews and Meta-Analyses extension for Scoping Reviews (PRISMA-ScR) Checklist. (PDF)

S1 Table Search query for PubMed. (PDF)

S2 Table Exclusion Criteria and their application. (PDF)

S3 Table Main characteristics of included studies on VC interventions for *Aedes aegypti* and *Aedes albopictus* in the sub-Saharan Africa region. Legend: BI, Breteau index – Bti, *Bacillus thuringiensis israelensis* – CBI, community-based intervention – CHIKV, Chikungunya virus – CI, Container index – CI 95%, 95% Confidence interval – DENV, Dengue virus – DF, Dengue fever – DHF, Dengue haemorrhagic fever – Entomol, entomological – Environ, environmental – Epidemiol, epidemiological – HI, House index – IgG, immunoglobuline G – ITN, Insecticide-treated net – LISA, Local Indicators of Spatial Association – LLIN, Long lasting insecticidal net – m, meters – mo, month – N, number – OR, Odds Ratio – PI, pupae index – P/PI, pupal/person index – RCT, randomised controlled trial – ULV, Ultra-low volume – y, year – YF, Yellow Fever.
(PDF)
