# Supplementary material for: Vector control for Aedes aegypti and Aedes albopictus mosquitoes implemented in the field in sub-Saharan Africa: A scoping review
Source: PLoS Negl Trop Dis. 2025 Jul 9;19(7):e0013203. doi: 10.1371/journal.pntd.0013203 (PMC12240363; doi:10.1371/journal.pntd.0013203)
Supplement: S1 Table — (PDF) [file pntd.0013203.s002.pdf]

Terms connected by *OR*	*AND*	Terms connected by *OR*
Setting		Population
(africa[All fields] OR afrique[All fields] OR africa south of the sahara[MeSH Terms] OR algeria[All fields] OR algerie[All fields] OR angola[All fields] OR benin[All fields] OR botswana[All fields] OR burkina faso[All fields] OR burundi[All fields] OR cabo verde[All fields] OR Cape verde[All fields] OR cameroon[All fields] OR central african republic[All fields] OR république centrafricaine[All fields] OR chad[All fields] OR comoros[All fields] OR congo[MeSH Terms] OR zaire[All fields] OR democratic republic of the congo[All fields] OR république démocratique du Congo[All fields] OR republic of the Congo[All fields] OR république du Congo[All fields] OR côte d'Ivoire[All fields] OR ivory coast[All fields] OR djibouti[All Fields] OR equatorial guinea[All fields] OR eritrea[All fields] OR eswatini[All fields] OR ethiopia[All fields] OR gabon[All fields] OR gambia[All fields] OR ghana[All fields] OR guinea[All fields] OR guinea-bissau[All fields] OR kenya[All fields] OR lesotho[All fields] OR liberia[All fields] OR madagascar[All fields] OR malawi[All fields] OR mali[All fields] OR mauritania[All fields] OR mauritius[All fields] OR mozambique[All fields] OR moçambique[All fields] OR namibia [All fields] OR niger[All fields] OR nigeria[All fields] OR rwanda[All fields] OR sao tome and principe[All fields] OR senegal[All fields] OR seychelles[All fields] OR sierra leone[All fields] OR south africa[All fields] OR south sudan[All fields] OR sudan[All fields] OR somalia[All fields] OR tanzania[All fields] OR togo[All fields] OR uganda[All fields] OR zambia[All fields] OR zimbabwe[All fields])		(aedes[All fields] OR aegypti[All fields] OR albopictus[All fields] OR Stegomyia[All fields] OR albopicta[All fields])

S1 Table. Search query for PubMed
