# Supplementary material for: Vector control for Aedes aegypti and Aedes albopictus mosquitoes implemented in the field in sub-Saharan Africa: A scoping review
Source: PLoS Negl Trop Dis. 2025 Jul 9;19(7):e0013203. doi: 10.1371/journal.pntd.0013203 (PMC12240363; doi:10.1371/journal.pntd.0013203)
Supplement: S2 Table — (PDF) [file pntd.0013203.s003.pdf]

EXCLUSION CRITERIA	APPLICATION of EXCLUSION CRITERIA
• Referred to elsewhere than in SSA<br>• Referred to other mosquito species than *A. aegypti* and/*or A. albopictus*<br>• Not presenting any vector control method<br>• The vector control method is not implemented in the field (e.g. laboratory experiments)<br>• No measures of entomological and/or epidemiological endpoints are provided<br>• Published before 2000	• In the absence of any of the previous exclusion criteria, the record is included in the screening based on title/abstract and in the full-text evaluation (type IN)<br>• If there is at least one of the preceding exclusion criteria, the record is excluded (type OUT) with explanation of the reason of exclusion<br>• The first exclusion criteria identified, if any, is sufficient to exclude the record with no need to continue with checking for subsequent exclusion criteria.<br>• At the stage of screening based on title/abstract, if there is doubt, the record is included for full-text evaluation (type IN)<br>• In case of discordance, agreement may be found through discussion between the two evaluators first and, if not, by consulting a senior academic researcher

S2 Table. Exclusion Criteria and their application
