# Supplementary material for: Vector control for Aedes aegypti and Aedes albopictus mosquitoes implemented in the field in sub-Saharan Africa: A scoping review
Source: PLoS Negl Trop Dis. 2025 Jul 9;19(7):e0013203. doi: 10.1371/journal.pntd.0013203 (PMC12240363; doi:10.1371/journal.pntd.0013203)
Supplement: S3 Table — Legend: BI, Breteau index – Bti, Bacillus thuringiensis israelensis – CBI, community-based intervention – CHIKV, Chikungunya virus – CI, Container index – CI 95%, 95% Confidence interval – DENV, Dengue virus – DF, Dengue fever – DHF, Dengue haemorrhagic fever – Entomol, entomological – Environ, environmental – Epidemiol, epidemiological – HI, House index – IgG, immunoglobuline G – ITN, Insecticide-treated net – LISA, Local Indicators of Spatial Association – LLIN, Long lasting insecticidal net – m, meters – mo, month – N, number – OR, Odds Ratio – PI, pupae index – P/PI, pupal/person index – RCT, randomised controlled trial – ULV, Ultra-low volume – y, year – YF, Yellow Fever. (PDF) [file pntd.0013203.s004.pdf]

S3 Table. Main characteristics of included studies on VC interventions for *Aedes aegypti* and *Aedes albopictus* in the sub-Saharan Africa region

| | Country, Author, Year | Objective (s) | Study design | Intervention(s) | Indicator(s) | Main reported findings |
|---|---|---|---|---|---|---|
| 1 | Sudan, Seidahmed, 2012 [23] | To evaluate the impact of an integrated vector control response plan during a major DENV outbreak in a Red Sea coastal area in Sudan in 2010 | Pre-post (before-after) study<br><br>IMPLEMENTATION*<br>w10 (Mar2010) to w21 (May2010)<br>EVALUATION<br>Entomol, w9 to w22<br>Epidemiol, w7 to w31<br>(Evaluation regularly performed using weekly reports of the vector surveillance and spot checks of entomological surveys and supervisory visit)<br><br>** Times refer to the epidemiological weeks (w) of the outbreak* | • Community mobilization (scrubbing and drying of unused containers by householders)<br>• Indoor and outdoor thermal fogging and ULV sprays of permethrin<br>• Chemical larviciding with temephos of outdoor container<br>• Distribution of LLINs to in- and out-patients<br>• Advice on daytime use of repellents | • Entomol (CI, HI, BI, P/PI)<br>• Epidemiol (N of human DENV cases based on IgM/IgG RDT, NS1 ELISA and IgM ELISA assays + PCR on a subsample) | • Entomol indices were higher in the middle sector compared to the southern and eastern sectors<br>• Overall infested indoor water storage containers (22%) : clay pots (75%) > plastic barrels (15%)<br>• Significant reduction of HI (from 100% to 16%, F = 57.8, P < 0.001) and P/PI (from 0.77 to 0.10 in week 21, F = 3.06, P < 0.01)<br>• The coverage rate of community mobilization was > 70%<br>• Reduction of human DENV incidence rate from 9 cases/10 000 (341 new cases) to zero incidence with an overall outbreak attack rate of 94 cases/10 000 (3765 DF/DHF cases) higher in eastern and southern sectors (OR = 2.44 and 2.07 respectively) compared to the middle sector (OR = 1.88)<br>• By regression analysis, a significant relationship was found between the entomol parameters and dengue incidence over the weeks of surveillance (R2 = 0.83, F = 23.9, P < 0.001). |
| 2 | Ethiopia, Waldetensai, 2021 [24] | To evaluate the impact of environmental management and chemicals (temephos and propoxur) on *Aedes* mosquitoes to control the CHIKV outbreak in Dire Dawa, Ethiopia, in 2019 | Pre-post (before-after) study<br><br>IMPLEMENTATION<br>Aug2019 to Oct2019<br>EVALUATION<br>Pre-, Aug2019<br>Post-, w1 after implementation | • Chemical indoor and outdoor space spraying of propoxur<br>• Chemical larvicides temephos in stored water<br>• Environ management<br>• Community education | • Entomol (N of adult mosquitoes, HI, CI, BI, PI) | • Outdoor clean water containers the most infested : tyre (25.2%) > barrel (17.8%) > flower pot (16.9%) > jerrycan (15.1%)<br>• After the intervention, *Aedes* adult mosquitoes reduced in all collected resting sites at the day time (P = 0.031)<br>• Reduction of CI (from 92.9% to 14.7%), BI (from 141 to 20.1), HI (from 90.1% to 7.4%) and PI (from 1431.4 to 4.12) |

| | Country, Author, Year | Objective (s) | Study design | Intervention(s) | Indicator(s) | Main reported findings |
|---|---|---|---|---|---|---|
| 3 | Burkina Faso, Ouédraogo, 2018 [25] | To evaluate the effectiveness of a CBI for DENV vector control in Ouagadougou, Burkina Faso | Cluster-RCT<br><br>IMPLEMENTATION<br>Jun2016 to Oct2016<br>EVALUATION<br>Pre-, Oct2015<br>Post-, Oct2016 | • Community education for behaviour change (including education on DENV infection, transmission and prevention) / Environ management (including public space clean-up activities) | • Epidemiol (human biomarkers for exposure to *Aedes aegypti* mosquito)[a]<br>• Entomol (CI, HI, BI, PI, N of water breeding sites*, N of containers with larvae/ pupae*, N of larvae*, N of pupae*)[a]<br>• Knowledge (self-reported)[b]<br>• Attitudes and practices (self-reported)[b]<br><br>*[a] Primary outcomes evaluated at the compound level (≥1 households sharing the same living space)*<br>**At house level*<br>*[b] Secondary outcomes evaluated at the household level* | • By regression analysis, the intervention reduced exposure to *Aedes aegypti* mosquito bites (coefficient –0.08 [95% CI –0.11 to –0.04])<br>• CI, HI, BI, PI : all reduced in the intervention arm and little or no change or increase in the control arm<br>• By regression model, the intervention did not show an effect on the absolute N of *Aedes aegypti* mosquito breeding sites or on the N of larvae/pupae at the compound level (no calculated difference of the intervention effect was statistically significant)<br>• In the intervention arm, increase in DENV knowledge (risk ratio [RR] 1.13 [95% CI 1.01–1.27]), disease symptoms identification (RR 1.44 [95% CI 1.22–1.69]) and reduced association of DENV infection with malaria RR 0.70 [95% CI 0.58–0.84]<br>• In the intervention arm, increase in self-reported actions against mosquitoes (RR 1.42 [95% CI 1.29–1.57]) and bed nets use (RR 1.31 [95% CI 1.22–1.42]) |
| 4 | Burkina Faso, Bonnet, 2020 [26] | Through spatial analysis methods, to determine the spatial distribution of a CBI against *Aedes aegypti* mosquitoes and to assess where the | Case study based on the previous cluster-RCT (*Ouédraogo, 2018)* | As illustrated in *Ouédraogo, 2018* | • Entomol (CI, HI, BI, P/PI, N of immature instars, N of larvae per inhabitant at household level representing the N | • Discarded containers were the most infested (68.8% and 62.4% in control and intervention arm respectively) followed by containers for water storage (27.5 and 36.5% respectively). After the CBI, in the intervention group, water storage recipients were reduced (-69.4%) but not the discarded containers (+5.7%)<br>• CI, HI, BI, PI : as *Ouédraogo, 2018* |

| | Country, Author, Year | Objective (s) | Study design | Intervention(s) | Indicator(s) | Main reported findings |
|---|---|---|---|---|---|---|
| | | intervention was most effective | | | of positive breeding sites) | • After the intervention, immature stages were significantly fewer in the intervention arm than in the control (t = 2.362; P = 0.0186)<br>• The average difference of the proportion of positive containers between the intervention and control arms (calculated as within-household change before-after, for 242 households) was 9.67% (95% CI: 1.1–18.3%)<br>• Spatial analysis showed that after the intervention, the number of concentration areas of high and low values of pupae was reduced in the intervention arm while aggregates persist in the same places or nearby in the control arm |
| 5 | Kenya, Forsyth, 2022 [27] | To design a source reduction intervention targeted at caregivers and children and evaluate its effectiveness in improving source reduction knowledge and behaviours and subsequent changes in entomological indices in coastal Kenya | Matched-pair cluster RCT<br><br>BEFORE IMPLEMENTATION<br>Stakeholder workshop , Nov2016<br>Pilot study, Jan2017 to Mar2017<br>IMPLEMENTATION<br>May2017 to Jul2017<br>EVALUATION<br>Pre-, at baseline<br>Post-, at mo3 (except for observed behaviors), at mo12, from mo12 to mo15 (Qual evaluation only for 34 purposively selected caregivers)<br>EXTENSION of the intervention to the | • CBI for source reduction behaviours (including covering containers, removing trash and unused containers, moving containers out of the rain, and removing or poking holes in tires) | • Knowledge[a]<br>• Behaviours (self-reported and observed)[a]<br>• Entomol (CI, HI, N of containers/household)[b]<br>• Qual evaluation (to understand barriers and facilitators to behaviour change)<br><br>*[a]Primary outcomes*<br>*[b]Secondary outcomes* | • Knowledge increased and higher in the intervention arm at mo3 and mo12, reaching >50% (adj risk difference 0.69, 95% CI [0.56 to 0.82])<br>• Self-reported behavior improved in the intervention arm at mo3 and mo12, reaching >50% (adj risk difference was 0.58, 95% CI [0.43 to 0.73]). The 2 most common behaviors: *'covering containers'* (60% in the intervention vs 10% in the control) and *'move containers out of rain'* (39% vs 2%)<br>• Observed behaviors showed no significant difference between intervention and control at mo12 : behavior *'at least 1 covered container'* adj risk difference -0.08, 95% CI [-0.13, -0.01] and behavior *'total N of container habitats'* adj mean difference 0.05, 95% CI [-0.98, 0.68]<br>• The type of containers for most immature mosquitoes were laundry recipients > containers with no purpose > for sanitation in the intervention, while in the control arm, containers with no purpose (>60%) > laundry recipients<br>• CI and HI showed no difference at mo3 months between control and intervention (respectively adj mean difference -0.01, 95% CI [-0.04, 0.02] and adj risk difference 0.01, 95% CI [-0.03, 0.06] |

| | Country, Author, Year | Objective (s) | Study design | Intervention(s) | Indicator(s) | Main reported findings |
|---|---|---|---|---|---|---|
| | | | control arm at the end of y2018 | | | • In the qualitative evaluation at mo3, *'Intention to cover'* was the most frequent behavior and *'Move containers'* was higher in adopters than non-adopters. Benefit from the intervention was higher in adopters than non-adopters. Among referred barriers: interference from others (children playing), high number of containers, losing covers. Among facilitators: concerns about hygiene, disease prevention, clean and safe water |
| 6 | Burkina Faso, Dambach, 2021 [28] | To evaluate the impact of biological antimalarial larviciding with Bti on non-malarial mosquitoes in a rural and a semi-urban setting in Burkina Faso | Cluster-RCT<br>Three study arms (9 village clusters randomly assigned)<br>i) control-untreated<br>ii) Bti100%-treatment of all breeding sites<br>iii) Bti50%-risk map based larvicide application<br><br>IMPLEMENTATION<br>Two years, 2014 (data available) and 2015 (data not available)<br>EVALUATION<br>Pre-, Sep2013 to Dec2013<br>Post-, Jun2014 to Nov2014 (adult mosquito monitoring every two weeks) | • After identification of optimal larvicidal dosages for field, Bti spraying every ten days followed the day after by a quality control test by dipping for live larvae, for up to six weeks after the rainy season in villages (in public spaces only, excluding private premises) and in a 500 m buffer zone around villages | • Entomol (mosquito abundance by indoor and outdoor light traps) | • *Aedes* capture predominantly indoor at 57% (p = 0.071)<br>• *Aedes* share unchanged in 2013 (2.317 - 19%) vs 2014 (5.357 - 22%)<br>• *Aedes* mosquito abundance significantly reduced by 34% (vs 70% reduction of A*nopheles*) in the Bti100%-arm (rate ratio RR 0.66, 95% CI: 0.57–0.76) but not in the Bti50%-arm (RR 0.94, 95% CI: 0.85–1.05)<br>• Major impact of the intervention was in the semi-urban town > in the rural study villages and during Aug, rise in *Aedes* N in Sep and Oct |
| 7 | Gabon, Gabor, 2016 [29] | To assess seropositivity for IgG anti-CHIKV and anti-DENV and its | Retrospective cohort study | • ITNs | • Epidemiol (anti-CHIKV IgG and anti-DENV IgG** | • Increase in DENV seropositivity (from 1.2% to 12.3%), possible DENV infection in 13 children. In a proportion of |

| | Country, Author, Year | Objective (s) | Study design | Intervention(s) | Indicator(s) | Main reported findings |
|---|---|---|---|---|---|---|
| | | association with ITN use, prior to outbreaks in Gabonese children | IMPLEMENTATION<br>Dec2002 to Apr2007 | | seroprevalence, ITNs use, vaccination status, including YF)<br><br>*** The DENV-positive results were correlated with the time points of YF vaccination and signs and symptoms compatible with DENV infection* | cases, interpretation of individual DENV serology results was vague<br>• Stable CHIKV seropositivity (0.6%)<br>• Decrease in the ITNs (96% to 79%)<br>• No correlation between reported ITNs use and DENV/CHIKV seropositivity |
| 8 | Ivory Coast, Kone, 2005 [30]<br><br>*Article in French* | To evaluate the effect of spatial spraying with deltamethrin on *Aedes aegypti* mosquito population in two municipalities (maritime and forestry) in Ivory Coast | Pre-post (before-after) study<br><br>IMPLEMENTATION<br>May1997<br>EVALUATION<br>Pre-, May1997 (before the first ULV application)<br>Post-, i) right after the first application and before the second and ii) five days after the second application | • Two ULV applications of deltamethrine one week apart | • Entomol (mosquito abundance by indoor and outdoor human capture) | • *Aedes aegypti* abundance reduced in both sites after the first ULV application: maritime 37.5% (69% reduction of global density) vs forest 66% (15% reduction of global density)<br>• Bites/human/sampling period: maritime 5.58 vs forest 2.5<br>• *Aedes aegypti* endophagic behavior : maritime 69% vs forest 11%<br>• *Aedes aegypti* abundance back to the pre-intervention values in both sites 5 days after the second ULV application (vs 49% reduction of global density in the forest site while unmodified in the maritime site) |

**Legend:** BI, Breteau index – Bti, *Bacillus thuringiensis israelensis* – CBI, community-based intervention – CHIKV, Chikungunya virus – CI, Container index – CI 95%, 95% Confidence interval – DENV, Dengue virus – DF, Dengue fever – DHF, Dengue haemorrhagic fever – Entomol, entomological – Environ, environmental – Epidemiol, epidemiological – HI, House index – IgG, immunoglobuline G – ITN, Insecticide-treated net – LISA, Local Indicators of Spatial Association – LLIN, Long lasting insecticidal net – m, meters – mo, month – N, number – OR, Odds Ratio – PI, pupae index – P/PI, pupal/person index – RCT, randomised controlled trial – ULV, Ultra-low volume – y, year – YF, Yellow Fever
